# Fat Content and Composition in Retail Samples of Australian Beef Mince

**DOI:** 10.3390/nu6062217

**Published:** 2014-06-11

**Authors:** Flavia Fayet-Moore, Judy Cunningham, Tim Stobaus, Veronique Droulez

**Affiliations:** 1 Nutrition Research Australia, Level 13, 167 Macquarie St, Sydney, NSW 2000, Australia; E-Mail: flavia@nraus.com; 2 Food Standards Australian New Zealand, 55 Boeing House 55 Blackall Street, Barton, ACT 2600, Australia; E-Mail: judy.cunningham@foodstandards.gov.au; 3 National Measurement Institute, Department of Industry, Innovation, Science, Research and Tertiary Education 153 Bertie Street, Port Melbourne, VIC 3207, Australia; E-Mail: tim.stobaus@measurement.gov.au; 4 Meat & Livestock Australia, 40 Mount Street, North Sydney, NSW 2060, Australia

**Keywords:** fat, beef, mince, composition, retail, variability, Australian, red meat

## Abstract

Nutrient composition data, representative of the retail supply, is required to support labelling and dietetic practice. Because beef mince represents approximately 30% of all beef dishes prepared in Australian households, a national survey of the different types of mince available for purchase in representative retail outlets was conducted. Sixty-one samples of beef mince from 24 retail outlets in New South Wales, Queensland, Victoria and Western Australia were collected in 2010 and analysed for moisture, protein, total fat and fatty acid profile. A variety of 18 different descriptors were used at point of sale with “Premium” (*n* = 15) and “Regular” (*n* = 8) the most commonly used terms. The analysed fat content of “Premium” samples varied from 2.2 g/100 g to 8.0 g/100 g. Forty-eight percent (*n* = 29) of the samples were categorised as low fat (<5 g/100 g; mean 4.1 g/100 g), 21% as medium fat (5–10 g/100 g; mean 8.9 g/100 g) and 31% as high fat (>10 g/100 g; mean 10.4 g/100 g). There was no significant difference between the types of mince available for purchase in low *versus* high socio-economic suburbs (Chi-square, *p* > 0.05). In conclusion, the fat content of the majority of retail beef mince in Australia is <10 g/100 g and a variety of descriptors are used at point of sale, all of which do not necessarily reflect analysed fat content.

## 1. Introduction

Beef mince is commonly consumed in Australia, representing approximately 30% of all beef dishes served [1]. Up-to-date nutrient composition data representative of beef mince as typically consumed by Australians is required to support labelling, nutrition research and dietetic practice.

Food Standards Australia and New Zealand’s food and nutrient database, NUTTAB2010 [2], contains nutrient data for 2668 foods available in Australia, including beef mince. There are two main types of fat-related descriptors used to reflect types of beef mince in the nutrient database. NUTTAB2006 [3], the most recent database at the time of this study, provided data using four types of beef mince descriptors: (i) hamburger; (ii) regular; (iii) premium and (iv) low fat. The current version, NUTTAB2010 provides data for two types of beef mince: (i) low fat including lean or heart smart and (ii) regular. The main difference between the different descriptors used to describe beef mince was their total fat content. In NUTTAB2006, total fat content varied from 12.1 g/100 g in “hamburger” mince to 10.8–12.9 g/100 g in “regular” mince, 9.9 g/100 g in “premium” mince and 7–9.9 g/100 g in “low fat” mince. 

Analyses of individual samples prepared by different butchers showed variability within samples when prepared according to the same specifications. For instance, the fat content of lean samples ranged from 6 g/100 g to 9 g/100 g [4]. There are many factors that can influence total fat content of beef mince, including the type of beef cuts or trim used to prepare the mince and the choice of point of sale descriptor used by the butcher or retail outlet.

Therefore, a sampling plan that captures the variability in fat content of beef mince is needed to ensure data accurately reflect beef mince available for purchase in Australia. Current protocols for analysing the nutrient composition of red meat have been based on homogenates derived from 10 samples randomly selected from retail outlets [5,6,7,8]. Unpublished analyses from individual samples suggest this protocol may not adequately capture the variability within samples of the same specification available for purchase in the retail supply [9].

The aim of this study was to determine the total fat content and fatty acid profile of beef mince available for purchase in Australia utilising a sampling program which better represents retail supply variability by selecting a greater number of samples from key retail outlets supplying beef mince in Australia, including butchers and supermarkets located in different socio-economic suburbs and for supermarkets, both centralized and store-based preparation systems. 

## 2. Experimental Section

A national survey of the different types of beef mince available for purchase from 24 representative retail outlets in Australia was conducted.

### 2.1. Sampling

Sixty-one samples of beef mince were purchased from 24 retail outlets across four Australian states: New South Wales (NSW), Queensland (QLD), Victoria (VIC) and Western Australia (WA) in August 2010. Retail outlets were selected to reflect differences between the major types of outlets from which Australians purchase beef mince.

Samples were selected from a butcher and a supermarket located in a high and low socio-economic suburb in each state, representing a total of 24 retail outlets. High and low socio-economic status suburbs were determined using the Index of Relative Disadvantage classification system of the Australian Bureau of Statistics [10]. Retail outlets were randomly selected within each state and identified socio-economic suburbs. In addition, samples from four retail outlets (one in WA and three in NSW) where mince is centrally prepared and then distributed for sale in other stores were collected. 

From each of the retail outlets, 500 g of each type of beef mince available for purchase were purchased anonymously. The following information was documented during the collection of samples: the number of different types of mince available for purchase, the descriptor used to label mince at point of purchase, and the proportion of the retail case allocated to each type of mince. Information was obtained from each retailer regarding type of cut and level of trim used to prepare the beef mince. Mixed meat minces (such as veal and beef or beef and lamb) were excluded.

### 2.2. Sample Preparation

Upon collection, each 500 g sample of raw mince was placed in a labelled plastic bag and stored in cold storage containers to prevent moisture and nutrient losses. Samples were transported to the National Measurement Institute (NMI, Melbourne, Australia) in a chilled condition within 24 hours of purchase. Raw samples were immediately homogenised in a heavy-duty blender and stored in plastic sample containers with screw top lids. The containers were filled to a minimum headspace and stored at −18 °C prior to analysis. Each container was labelled with a sample description and a unique Laboratory Registration Number.

### 2.3. Analytical Methods

Homogenates were thawed and each sample was analysed for total fat (soxhlet extraction, NMI in house method VL300, based on AOAC 960.39) and fatty acid profile by gas chromatography (NMI in house method VL289). In this method, fat was extracted using a combination of methanol and chloroform and then methylated by sodium methoxide; fatty acid methyl esters were separated using a Agilent 6890 Gas Chromatograph using a 100 m Supelco SP-2560 capillary column with Flame Ionisation detection. These methods are the same as those stated in Williams *et al.* [8].

Approximately eight months post initial sampling further analysis was commissioned for cooked samples. Due to deterioration of some samples during storage, a small number (*n* = 14) of representative beef mince samples, excluding all samples from WA, were cooked and analysed. Mince was cooked on a non-stick frying pan for 3–5 min without the addition of fat.

### 2.4. Statistical Analysis

All beef mince samples collected were included in the calculation of the average total fat composition for each of the main types of mince purchased. The main types of beef mince available for purchase were categorised by total fat content according to the descriptor used at point of purchase. This was possible for samples where descriptors were suggestive of fat content. Where labelling was not indicative of fat content (*i.e.*, gourmet), beef mince was categorised according to the total fat content as measured by NMI. All samples were then categorised into low (<5 g/100 g), medium (5–10 g/100 g) or high (>10 g/100 g) total fat categories.

The weighted mean total fat content of 61 samples of raw beef mince samples and 14 cooked mince samples was determined for each fat category (low, medium and high). The weighting for each mince sample was based on the market share [1] of each major retailer outlet within each state (NSW, VIC, QLD and WA). Differences between fat categories and nutrient composition were determined using Chi-Square, and statistical significance was set at *p* < 0.05. SPSS for Macintosh Version 19.0 (version 19; SPSS Inc., Chicago, IL, USA) was used to carry out all statistical analyses.

## 3. Results

Table 1 lists the different descriptors used at point of purchase; the number of retail outlets using these different descriptors; and the average fat content of samples labelled accordingly. Eighteen different descriptors were used at point of sale to differentiate between the different types of mince available for purchase, including Best Mince; Diet; Extra Lean; Gourmet; Hamburger; Heart Smart; Lean; Premium; Premium grade; Regular; 3- 4- and 5-Star; Super Lean; Top Mince; Topside and Ultra Fine mince. Whilst some descriptors were suggestive of the total fat content of beef mince, such as “Lean”, other descriptors referred to its quality (*i.e.*, Ultra Fine or Top Mince) or its use (*i.e.*, Hamburger). “Premium” was the most commonly used descriptor at point of sale (*n* = 15). Despite “Premium” beef mince containing an average of 8.0 g/100 g of fat, fat content ranged from 2.2 g/100 g to 10 g/100 g. Similarly, descriptors such as “Best” and “Diet” were not indicative of the analysed fat content. To ensure the accuracy of the data, the measured fat content for samples with descriptors which were either not suggestive or indicative of the analysed fat content were used for categorisation purposes. These included “Premium”, “Regular”, “Ultra fine”, “Gourmet”, “Diet”, “Best mince” and “Topside”. The descriptors for the remaining samples were used to categorise the samples into low, medium and high fat categories.

Most retailers reported preparing mince on-site, mainly from off-cuts. Offcuts are meat and fat trimmings that remain from regular meat cuts prepared for sale according to customer specifications. These are used to make mince with different proportions of meat and fat trim combined, depending on the type of mince required. For example, low fat mince may have 95%–98% meat trim and 2%–5% fat trim. Some retailers reported using round or topside to make lower fat mince and chuck and gravy beef (also called shin, without bone) cuts for higher fat versions. Some reported trimming fat from the meat flesh before mincing. 

**Table 1:** Average fat content of retail beef mince by descriptors at point of purchase.

Mince descriptor used by retailer	Number of retail outlets using descriptor	Average fat content ** (g/100 g)
Super lean	2	2.2
Regular (lean)	1	2.5
Premium grade	1	3.4
Ultra fine	1	3.4
Top mince	1	3.5
Heart Smart	6	4.1
5 star	5	4.3
Lean	1	4.7
Extra lean	1	4.8
4 star	5	6.8
Premium *	15	8.0
Regular	8	9.1
Gourmet	3	9.5
Hamburger	3	9.7
Diet	1	10.1
3 star	5	11
Best mince	1	13
Topside	1	14

* Total fat ranged from 2.2 to 10 g/100 g; ** As measured by National Measurements Institute, Australia.

Of the 61 samples collected, 29 (48%) were categorised as low fat, 13 (21%) as medium fat and 19 (31%) as high fat using the methodology described. Labels corresponding to low fat samples included Gourmet; Hamburger; Heart Smart; Lean or variation; Premium; Regular; 5 star; Top Mince and Ultra Fine. Labels corresponding to medium fat mince included 4 star; Premium; Gourmet; and Regular. Labels corresponding to high fat samples included 3 star; Regular; Hamburger; Topside; Best mince; and Diet. Hence, mince labelled with terms such as “Premium” may be low or medium fat, “Regular” may be low, medium or high fat and “Hamburger” may be either low or high fat.

Only 26% of the samples (16 out of the 61 samples collected) used descriptors featured in NUTTAB2010 (Table 2). Five samples used the term “Lean” or variation of Lean, (Super lean, Extra lean), eight samples used the term “Regular” and three samples used the term “Hamburger”.

**Table 2:** Number of samples using descriptors indicated for beef mince in NUTTAB2010.

NUTTAB2010 *	Number of samples	Percent of samples (%)
Lean	5	2
Regular	8	13
Hamburger	3	5

* NUTTAB2010-Food Standards Australia New Zealand foods database, 2011.

Fourteen retailers offered three different types and 10 retailers offered two different types of mince available for purchase. Of retailers with three different types of mince on offer, 13 offered a low, a medium and a high fat option and one retailer offered one low and two high fat options. Of those with two different types of mince on offer, six retailers offered a low and a medium fat option, three retailers offered a low and a high fat option and one retailer offered two high fat options. 

Of all samples collected from retail outlets in low socio-economic suburbs, 34.4% were low fat; 33.1% were medium fat and 34.4% were high fat. Of samples collected from high socio-economic suburbs, 44.8% were low fat, 31.0% were medium fat and 24.1% were high fat. There was no significant difference between the types of mince available for purchase in low *versus* high socio-economic suburbs Table 3 (Chi-square, *p* > 0.05). The fat content of mince prepared in store (7.6 ± 1 g/100 g) did not significantly differ from that of mince prepared centrally (7.0 ± 1 g/100 g). 

**Table 3:** Availability of low, medium and high fat retail beef mince by socio-economic status.

Fat Content Category	Low SES (%)	High SES (%)
Low	34.4% (*n* = 11)	44.8% (*n* = 13)
Medium	31.3% (*n* = 10)	31.0% (*n* = 9)
High	34.4% (*n* = 11)	24.1% (*n* = 7)

Abbreviations: SES- socioeconomic status, as described by the Socio-economic Indexes for Areas, Australian Bureau of Statistics 2006; Chi-square by SES category, *p* > 0.05.

The average weighted fatty acid composition for low, medium and high fat mince is listed in Table 4. For raw mince, average fat for low fat was 4.1 low, g/100 g, medium fat was 8.9 fa g/100 g and high fat was 10.4 at g/100 g. For cooked mince, low fat had an average fat content of 8.1 and h g/100 g, medium fat 16.4 ow g/100 g and high fat 18.1 w f g/100 g. Raw and cooked mince on average, contained 3.4 and 5.7 g/100 g of saturated fatty acids and 0.07 and 0.1 g/100 g of total omega-3 fatty acids (including Linolenic acid C18:3w3), respectively. Both cooked and raw low-fat mince had a higher percentage of long-chain *n*-3 fatty acid docosapentaenoic acid (DPA) than high-fat mince. A higher level of omega-3 in the low fat mince is consistent with a higher proportion of DPA in muscle meat than meat fat [11].

**Table 4:** Nutrient composition of raw and cooked retail beef mince (mean ± SE).

Nutrient *	Raw Mince				Cooked Mince			
	Low fat	Medium fat	High fat	Total weighted mean	Low fat	Medium fat	High fat	Total weighted mean
Moisture (g/100 g)	71.0 ± 2.5	67.6 ± 4.3	65.0 ± 3.2	68.8 ± 4.4	58.5 ± 2.3	53.6 ± 5.9	49.5 ± 4.7	55.8 ± 4.9
Protein (g/100 g)	22.9 ± 1.5	22.4 ± 1.1	22.5 ± 1.3	22.7 ± 1.4	32.3 ± 1.8	29.5 ± 2.6	30.3 ± 0.92	31.3 ± 2.2
Total Fat (g/100 g)	4.1 ± 1.4	8.9 ± 3.9	10.4 ± 3.4	7.1 ± 4.0	8.1 ± 2.6	16.4 ± 4.5	18.1 ± 3.4	11.8 ± 5.5
Total SFA (%)	47.2 ± 1.5	47.0 ± 1.9	48.5 ± 3.9	47.6 ± 2.6	48.1 ± 1.6	48.9 ± 1.2	47.5 ± 0.70	48.2 ± 1.4
Total MUFA (%)	44.1 ± 2.4	45.4 ± 2.2	43.6 ± 4.2	44.3 ± 3.0	44.0 ± 1.5	44.2 ± 1.1	42.5 ± 0.5	44.3 ± 1.4
Total PUFA (%)	4.7 ± 0.88	3.3 ± 0.51	3.2 ± 0.61	3.9 ± 1.0	3.7 ± 1.4	2.3 ± 0.84	1.8 ± 0.34	3.1 ± 1.4
Total Mono TFA (%)	3.3 ± 0.82	3.4 ± 1.5	3.9 ± 1.6	3.5 ± 1.3	3.5 ± 0.96	4.1 ± 0.71	4.5 ± 1.0	3.8 ± 0.93
Total Poly TFA (%)	0.67 ± 0.29	0.79 ± 0.28	0.80 ± 0.26	0.73 ± 0.28	0.61 ± 0.10	0.50 ± 0.50	0.61 ± 0.21	0.58 ± 0.11
n-3 Fatty Acids (%)	1.3 ± 0.39	0.8 ± 0.25	1.0 ± 0.33	1.1 ± 0.39	1.11 ± 0.47	0.50 ± 0.18	0.38 ± 0.09	0.84 ± 0.50
C22:5w3 DPA (%)	0.44 ± 0.18	0.27 ± 0.11	0.29 ± 0.12	0.35 ± 0.17	0.38 ± 0.25	0.07 ± 0.11	0.0	0.24 ± 0.26
C22:6w3 DHA (%)	0.03 ± 0.04	0.0	0.01 ± 0.03	0.02 ± 0.04	0.01 ± 0.03	0.0	0.0	0.01 ± 0.03

Abbreviations: SFA- saturated fatty acids; MUFA-monounsaturated fatty acids; PUFA-polyunsaturated fatty acids; TFA-trans fatty acids; DPA-docosapenanoeic acid; DHA-docoheaxanoeic acid; * Results are weighted percentages expressed as grams per 100 g and as a percent (%) proportion of total fatty acids.

## 4. Discussion

The average fat content of Australian raw beef mince available for purchase from retail outlets in Australia was 4.1 g/100 g for low fat mince; 8.9 g/100 g for medium fat mince; and 10.4 g/100 g for high fat mince. Approximately 70% of retailers had all three types of mince available for purchase. Low fat mince was the most widely available for purchase and sold by all retailers. 

There were no significant differences in the fat content of beef mince available for purchase by socio-economic area and by location of preparation (*i.e.*, in-store *vs.* central distribution). These findings are consistent with others who found that the fat content of retail beef and lamb cuts did not vary systematically with either type of retail outlet or socioeconomic area [12,13].

The fatty acid composition of these retail meat samples, including the findings of measurable levels of long-chain PUFAS, is consistent with earlier studies of Australian beef [3]. 

This is the first study to document descriptors used at point of sale for each of the samples analysed. Choice of descriptor is often based on the retailer’s descriptor of customer needs and tends to be specific to the retailer [14]. Whilst some descriptors described the fat content of the mince, such as “Lean”, other descriptors referred to the quality or the intended use of the mince, such as “Premium” or “Hamburger”. Because it was difficult to categorise samples according to the three types of mince described in Australian nutrient composition tables (*i.e.*, lean, regular and hamburger mince), samples were categorised according to fat content, including low (<5 g/100 g); medium (5–10 g/100 g); and high (>10 g/100 g). Samples were allocated to the relevant category either according to their analysed fat content (where descriptors were not clearly suggestive or indicative of the analysed fat content) or by descriptor (where it was clearly suggestive of its low fat content).

The fat content of raw mince available for purchase in Australia reported in a retail survey conducted in 2002 [2] was slightly higher than the current analysis for low fat (6.8 for Lean *vs*. 4.1 g/100 g in this study) and high fat mince (16.4 g/100 g for hamburger mince *vs*. 10.4 g/100 g in this study), but similar for medium fat mince (8.7 and 10.8 g/100 g for “regular” and “premium” mince *vs.* 8.9 g/100 g for “medium” fat in this study. For cooked mince, differences were also reported: fat content in the low fat category of cooked mince was lower in this study compared to the 2002 study (8.1 *vs*. 9.0 g/100 g), while medium and high fat categories were higher: (16.4 g/100 g for “medium” fat *vs*. 9.9–12.7 g/100 g for “Premium” and “Regular” mince in 2002; 18.1 g/100 g for “high” fat *vs*. 12.1 g/100 g for “hamburger” mince in 2002).

Several reasons may explain the differences in fat content observed. It is possible that actual fat content of beef mince has changed since 2002. Increasing demand for leaner meat over the years by consumers has led to more trimming by butchers and consequently, a wider availability of lean beef and lamb available for purchase by retail outlets [15]. Differences also exist in the methodology used to obtain average nutrient composition of beef mince. In the 2002 study, nutrient composition was based on a homogenate of 10 samples randomly selected from 10 retail outlets in different socio-economic areas of Melbourne, VIC and Sydney, NSW. In this study, the average nutrient composition was calculated from the analysed nutrient content of 61 individual samples weighted by state and retailer and included several states. In addition, samples in the 2002 study were selected according to a descriptor at point of sale suggestive of fat content. In this study, it has been shown that the descriptor does not accurately reflect fat content and consequently, data for each mince category in the 2002 study may have been skewed due to the inclusion of samples with either higher or lower fat content than suggested by the descriptor. By analysing individual samples, it was possible to determine differences between the fat content of the different descriptors used at point of sale. This data are more in depth including analysis of individual samples; and weighting by retail market share.

In the US, the National Nutrient Database [16] classifies ground beef into six categories ranging in fat content (5%, 10%, 15%, 20%, 25% and 30%). The Canadian Nutrient File reports ground beef in four categories, which have specified maximum fat percentage targets, regulated by law [17]. The categories include: extra lean, lean, medium and regular with maximum allowed fat content of 10%, 17%, 23% and 30%, respectively. In Norway, the Norwegian Food Composition Table also classifies beef mince according to fat content (maximum of 6% fat or 14% fat) [18]. As the majority of mince available for purchase in Australia fall below 10 g/100 g of fat, Australian mince would be classified as extra lean by the Canadian system and as 10% lean by the American system. The average fat content of raw Australian mince (7.1 g/100 g) is in line with the fat content of extra lean ground beef in Canada (7.6 g/100 g) [17], well under the average content of beef mince in Denmark (16 g/100 g) [19], and lower than both the extra lean beef mince (9.6 g/100 g) and beef mince (16.2 g/100 g) reported by the UK Nutrient Databank [20].

**Figure 1:**
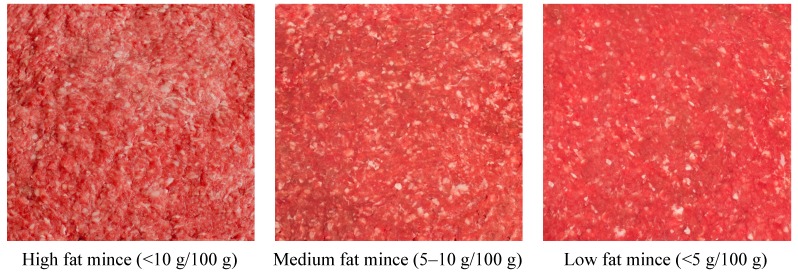
Visual representation of beef mince categorised by fat content.

In comparison to the United States and Canada, the labelling of fat content of mince (ground beef) is not regulated by law in Australia. It is not surprising that retailers use a wide range of types of descriptors of beef mince). Few of these feature in NUTTAB2010 as less than a third of samples (26%) collected used NUTTAB 2006/2010 descriptors. Therefore, these terms may not be reflective of mince available for purchase, making it difficult for users to select the most appropriate data. A small pilot study in Scotland compared total fat content of samples of minced beef sold and assessed the relationship between product label and total fat content [21]. In line with results of this study, there was wide variability in fat content by descriptor. “Minced beef”, descriptor ranged between 9.8% to 22.9% fat. It may therefore be more useful to categorise beef mince in nutrient databases into three categories based on their analysed fat content (low, medium and high). Examples of common descriptors, such as lean, 5 star and heart smart for low fat mince, could be provided. In addition, images illustrating raw mince for each of the fat categories (5 g/100 g; 5–10 g/100 g; >10 g/100 g), such as those in Figure 1, could be provided to assist users in selecting the most appropriate data. The lower the fat content, the less “white flecks” of fat are visible in the mince. 

Strengths of this study relate to an improvement in the accuracy of the data by including a larger sample size; analysis of individual samples; and the methodology used to classify samples according to analysed fat content. In addition, weighting data by retail market share took into account variability in mince available for purchase in Australia. A potential limitation of this study was that it did not reflect differences in nutrient composition due to seasonal variation. In lamb, long-chain omega-3 fatty acids were higher after spring in comparison to fall, primarily due to higher quality grass consumed in spring [22]. To our knowledge, the influence of seasonal variation on nutrient composition of beef in Australia has not been previously documented. Due to storage issues by NMI, not all samples were available to calculate cooked nutrient composition. Of the remaining samples, some nutrient losses may have occurred as samples were analysed eight months after the raw sample analysis. Despite weighting of the data, some states were not represented, including Victoria and South Australia, which may affect the values if there were major differences between these states. However, previous studies found little evidence of systematic differences in fat content between retail outlets and socio-economic regions [12,13].

## 5. Conclusions

There is a wide variability in the total fat content of retail beef mince in Australia. This study suggests that current sampling plans based on a homogenate of 10 random samples and categorised by descriptor at point of sale may not adequately represent this variability. Instead, sampling plans based on individual samples representative of retail market share and categorised by fat content is recommended to better capture this variability and improve the accuracy and consistency of data representing beef mince available for purchase in Australia.
